# Syndrome of progressive bone marrow failure and pancreatic insufficiency remains cryptic despite whole exome sequencing: variant of Shwachman‐Diamond syndrome or new condition?

**DOI:** 10.1002/ccr3.931

**Published:** 2017-04-04

**Authors:** Matthew C. Fadus, Eric T. Rush, Christina K. Lettieri

**Affiliations:** ^1^ Children's Hospital of PhiladelphiaPhiladelphiaPennsylvania; ^2^ Munroe‐Meyer Institute for Genetics and RehabilitationUniversity of Nebraska Medical CenterOmahaNebraska; ^3^ Children's Hospital and Medical CenterOmahaNebraska; ^4^ Department of PediatricsUniversity of Nebraska Medical CenterOmahaNebraska; ^5^ Kaiser PermanenteRosevilleCalifornia

**Keywords:** Anemia, Diamond‐Blackfan, Native American, Shwachman‐Diamond, thrombocytopenia

## Abstract

This case underscores the difficulty in diagnosis of bone marrow failure disorders, as the presentation of disease can be inconsistent, complicated by complex and ever‐expanding genetic etiologies. A patient who presents with bone marrow failure and pancreatic insufficiency raises the question of Shwachman‐Diamond syndrome (SDS) or a new condition which resembles SDS.

## Clinical Report

A Native American male from the Winnebago tribe was born at full‐term to a 21‐year‐old G1P0 mother by Caesarian section. He required a two‐day NICU stay for transient tachypnea of the newborn and hypoglycemia. The patient presented at 2 months of age with a two‐week history of decreased appetite, pallor, and watery diarrhea. There was no history of bleeding or bruising. Complete blood count showed hemoglobin of 1.9 g/dL (Normal for age: 9–13.5), white blood cell count of 11,500/mm^3^ (Normal for age 5–19.5), and platelet count of 161,000/mm^3^ (Normal: 140,000–450,000)**.** His reticulocyte count at admission was 5.72%. He was transfused to a hemoglobin of 8.2 g/dL. At discharge his leukocyte count was 6790/mm^3^ and platelet count was 117,000/mm^3^. Etiology of the anemia was not able to be determined during the initial inpatient hospitalization.

**Figure 1:**
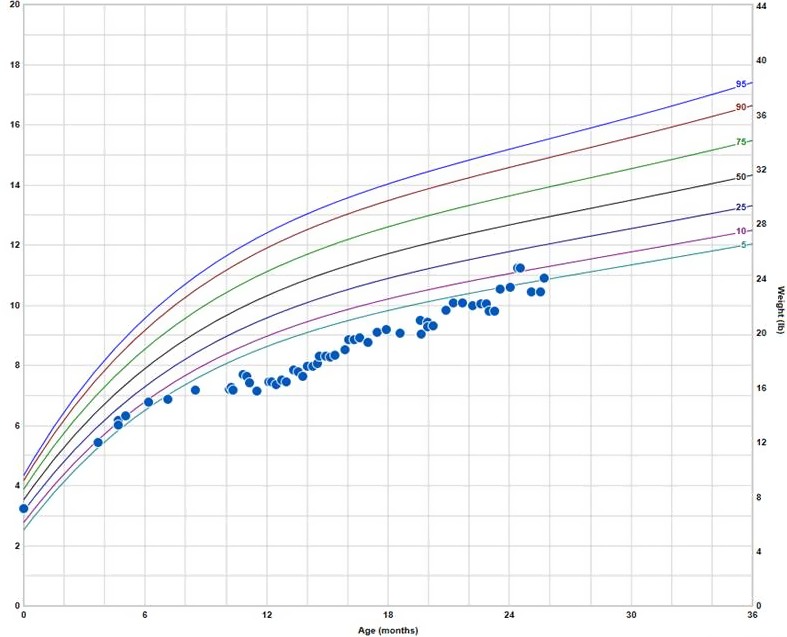
Growth Chart demonstrating poor growth for first 2 years.

At 3 months of age, he was found to be at the 1st percentile for height and 8th percentile for weight (see Fig. 1). At that time hemoglobin was 10.1 g/dL, leukocyte count was 4300/mm^3^, reticulocyte count was 1.24%, and platelet count was 180,000/mm^3^.

At 4 months of age, the patient returned for routine follow‐up. He was fussy, but did not have changes in stool or urine output or any recent infections. Hemoglobin was 7.1 g/dL, mean corpuscular volume (MCV) was 89.7 fL, and reticulocyte count was <0.5%.

Bone marrow aspirate was performed, and results showed normocellular bone marrow with erythroid aplasia, raising suspicion for Diamond‐Blackfan anemia. Infectious causes of red cell aplasia including, EBV, HIV, and CMV tests were negative. Parvovirus was IgG positive, IgM negative, consistent with maternally acquired antibodies or new infection. Iron, folate, and vitamin B12 levels were normal.

**Figure 2:**
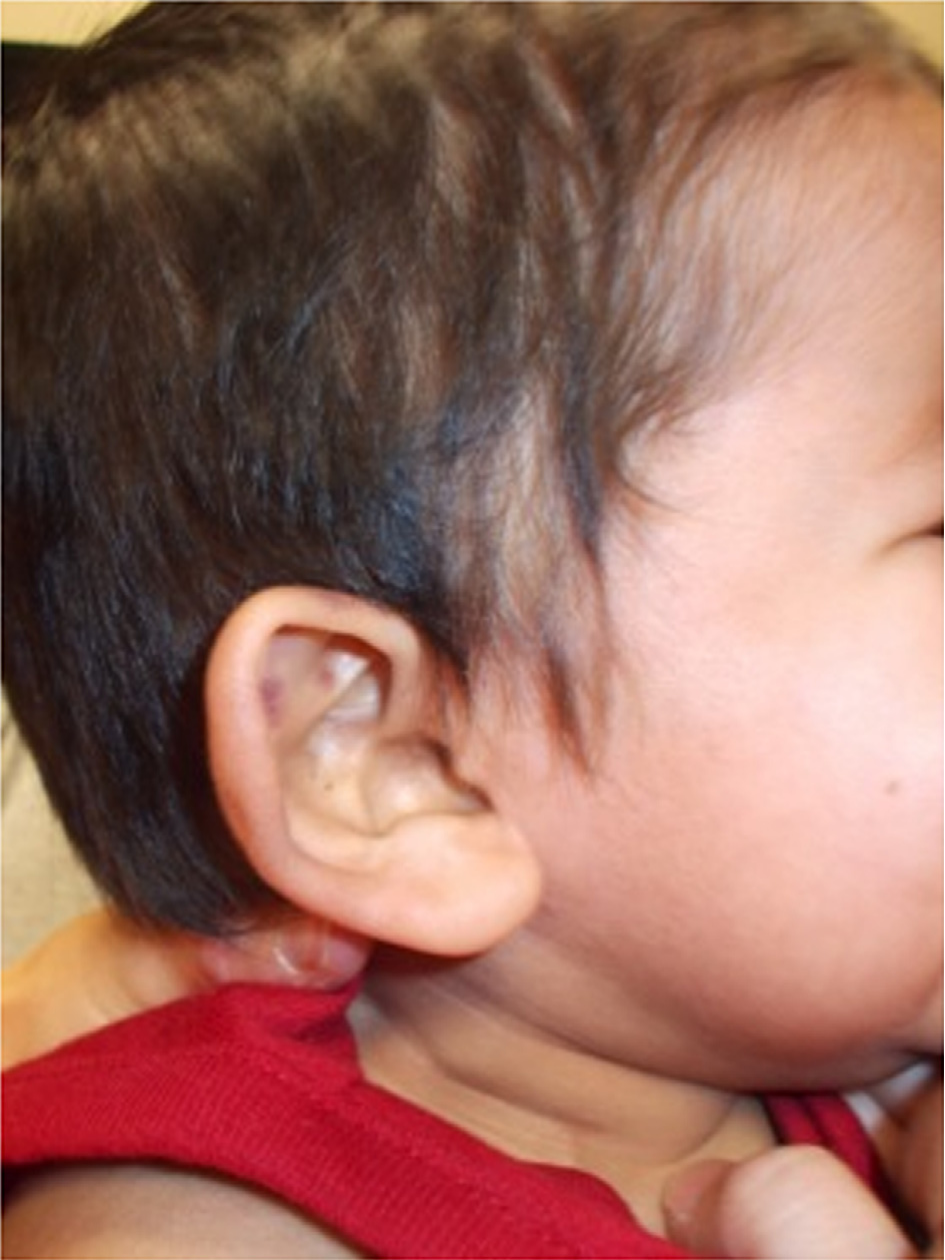
Dysplastic pinna showing cupping and poor development of both the helix and antihelix.

**Figure 3:**
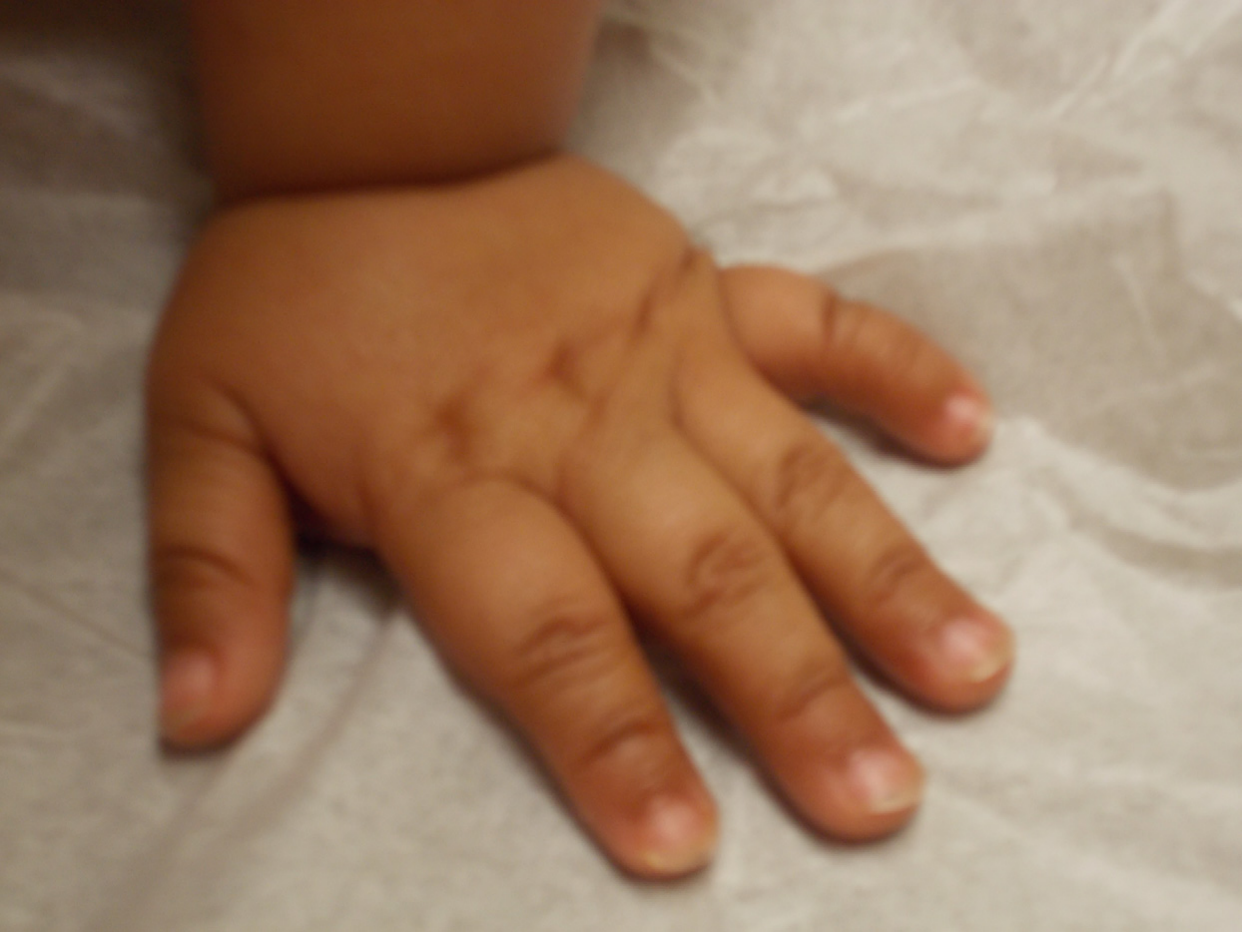
Small hands for age, with hypoplasia of the terminal phalanges on digits 2–5 with additional hypoplasia of the middle phalax on digit five resulting in clinodactyly.

At 6 months of age, his baseline hemoglobin was 9.5–10.5 g/dL, and he remained at the 3rd percentile for height and weight. His MCV ranged from 82.6 to 88.0 fL. Further examination was notable for a dysplastic right ear, which was small, cupped, with relatively poor helical and antihelical development (see Fig. 2). The left ear appeared normal. He also had small hands with appearance of small terminal phalanges, fifth finger clinodactyly and small feet (see Fig. 3). His platelet count began to decline to 80,000–100,000/mm^3^. Reticulocyte count continued to be low at 1.13%. Sequencing of genes implicated in Diamond‐Blackfan anemia was normal. Telomere length analysis for dyskeratosis congenita (Repeat Diagnostics, Vancouver, BC) and chromosome breakage analysis for Fanconi anemia (Human Genetics Laboratory, University of Nebraska, Omaha, NE) were normal.

At 10 months of age, hemoglobin remained stable at 9 g/dL, but platelet counts declined to <50,000/mm^3^. A repeat bone marrow biopsy was performed, and showed moderately to markedly hypocellular bone marrow (20–30% cellularity) with trilineal elements identified. Flow cytometry analysis was not suggestive of acute leukemia and FISH was not consistent myelodysplastic syndrome.

At eleven months of age, the patient was hospitalized for bronchiolitis. Platelet count was 22,000/mm^3^, leukocyte count was 3490/mm^3^, and hemoglobin was of 7.1 g/dL, with a reticulocyte count of 0.71%. He was transfused with packed red blood cells and platelets. During this hospitalization the mother noted that he was having foul‐smelling stools, which raised suspicion for pancreatic insufficiency. Pancreatic elastase was low at 37 *μ*g elastase/g stool (normal >200), suggesting pancreatic insufficiency. A low trypsinogen level of 2.6 ng/mL (normal 10–57) also supported pancreatic insufficiency.

Cytopenias and pancreatic insufficiency raised concern for Shwachman‐Diamond syndrome. Sequencing of the *SBDS* gene was performed and was normal. Pearson syndrome was also considered, but was thought to be less likely given the absence of sideroblastic anemia or clinical features of liver disease. High density SNP array analysis did not show a copy number variant, but showed several regions of homozygosity, encompassing 3.6% of his genome.

There is no family history of bleeding, hematologic disorders, or growth concerns. Both parents are of Winnebago Native American ancestry, but there is no known consanguinity.

**Figure 4:**
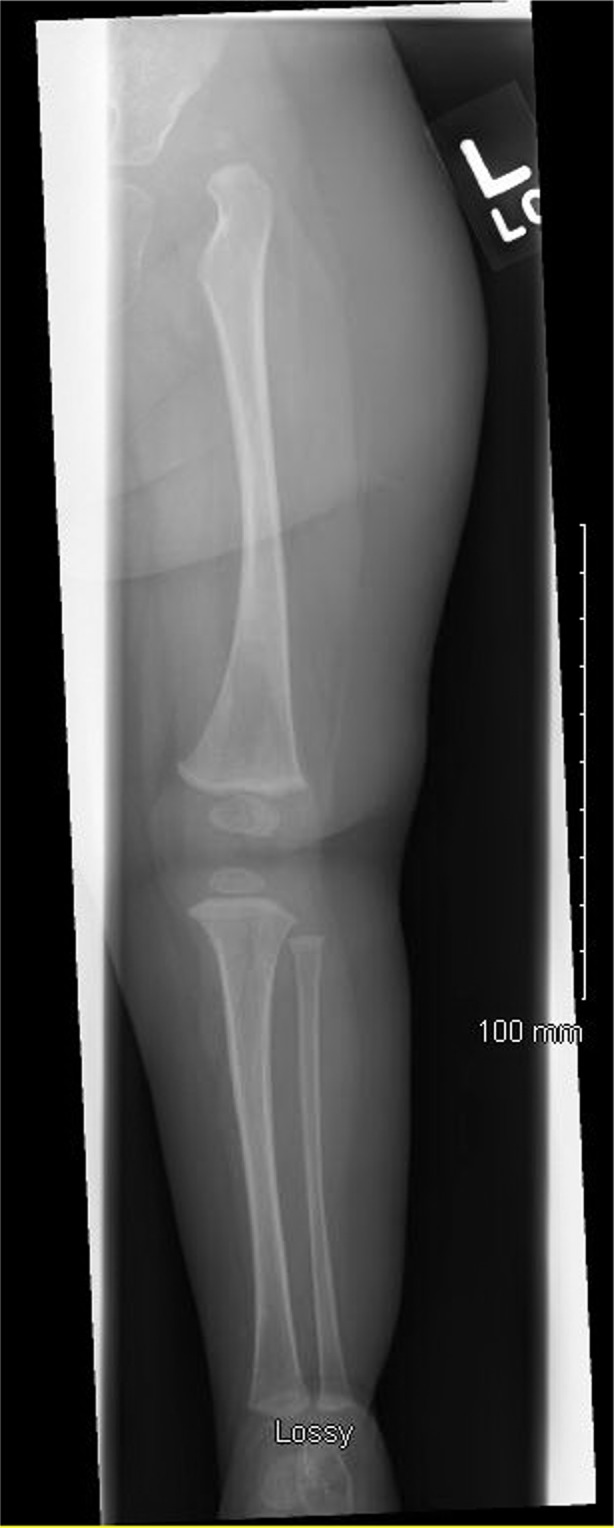
Left hip x‐ray demonstrating left hip dysplasia/complete dislocation.

Failure of targeted molecular testing to arrive at a definitive molecular diagnosis led clinicians to obtain whole exome sequencing (WES), which did not find variants which explained the patient's phenotype in the nuclear genome. Analysis of the mitochondrial genome showed no large deletions or pathogenic mutations. Analysis was limited for detection of novel genetic entities due to inability to obtain a paternal specimen. See genetic testing in Figure 4.

**Figure 5:**
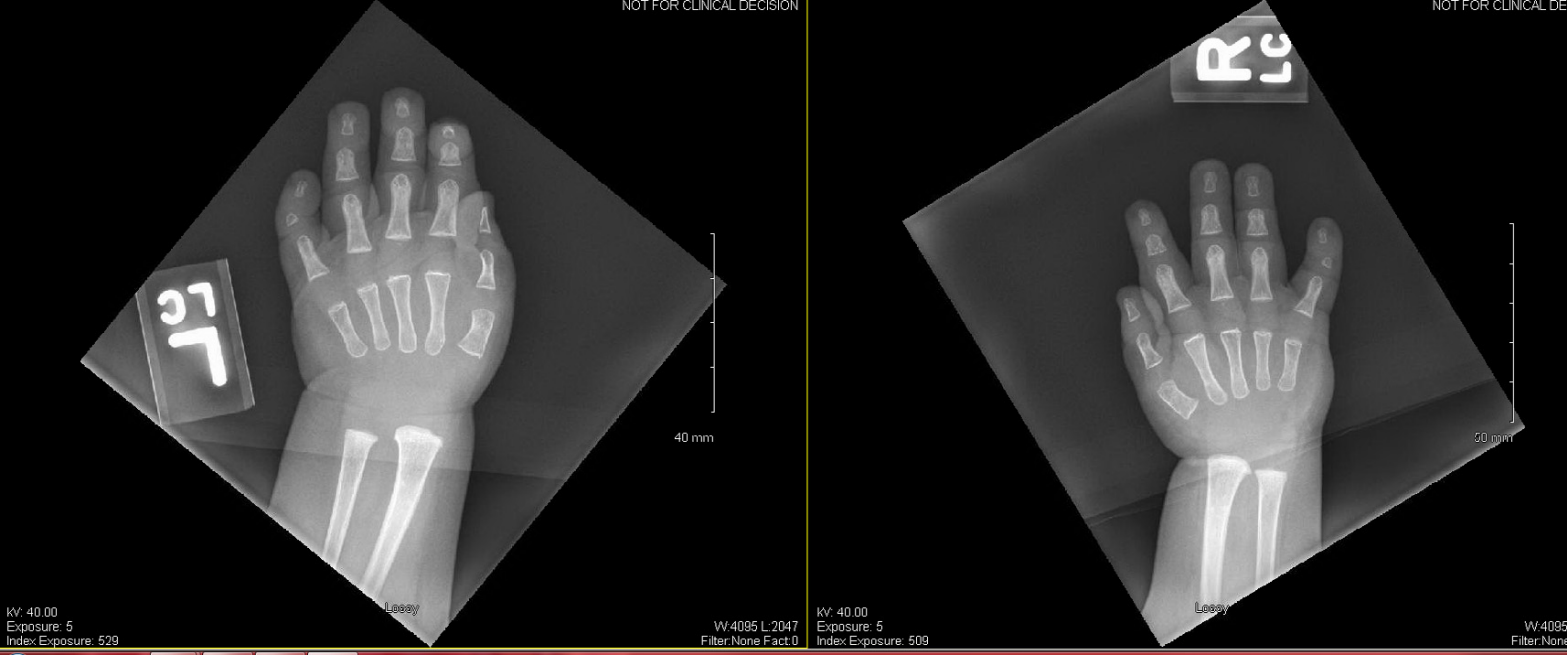
X‐rays demonstrating bilateral clinodactyly and bilateral hypoplasia of fifth middle phalanxy.

Echocardiogram and abdominal ultrasound were normal. Skeletal survey showed left hip dysplasia (Fig. 4), right coxa valga, and bilateral clinodactyly with hypoplasia of the fifth middle phalanx (Fig. 5).

The patient has been referred for hematopoietic stem cell transplant, but transplant has been unable to be performed due to lack of suitable matched unrelated donor. He is platelet transfusion dependent. Bone marrow aspirates and biopsies performed every 3–4 months continue to show hypocellurity (10–50% cellularity) without other diagnostic abnormality (Table 1).

**Table 1:** Genes tested

Syndrome tested	Laboratory	Date	Genes tested
Diamond‐Blackfan Anemia	Ambry Genetics	11/13/2013	RPL19, RPL26
Diamond‐Blackfan Anemia	Ambry Genetics	1/20/2014	RPS19, RPL5, RPL11, RPL35a, RPS26, RPS24, RPS17, RPS10, RPS7
Shwachman‐Diamond Syndrome	Ambry Genetics	4/16/2014	Full gene sequencing

## Discussion

This patient presents with pancreatic insufficiency and cytopenias, which prompted concern and evaluation for Shwachman‐Diamond syndrome although comprehensive analysis was not able to confirm this diagnosis or that of other conditions.

Shwachman‐Diamond syndrome (SDS) is a rare, autosomal recessive disease characterized by exocrine pancreas insufficiency, bone marrow failure, growth retardation, and skeletal dysplasia. SDS is the second leading cause of pancreatic exocrine insufficiency in children behind Cystic Fibrosis 1. Most patients develop symptoms of malabsorption and recurrent infection in the first 4–6 months of life. SDS has an estimated prevalence of 1/77,000 2. Over 90% of cases of SDS have been associated with a mutation in the SBDS gene, which is hypothesized to be involved in RNA processing and ribosomal structure 3. The etiology of the remaining 10% of SDS is largely unknown, although biallelic mutations in DNAJC21 have recently emerged as a cause of SDS 4.

All patients who have been reported with SDS are affected by some degree of exocrine pancreas dysfunction, as pancreatic acinar cells are replaced by fatty tissue. Uniquely in SDS, pancreatic function often improves with age. It is estimated that over 50% of patients with SDS will eventually cease requiring pancreatic enzyme replacement 5.

SDS is also characterized by bone marrow dysfunction in infancy. Neutropenia is the most common presentation, found in 98% of patients with SDS 1. Patients with SDS often present in early infancy with recurrent infections such as otitis media, pneumonia, sinusitis, and sepsis.

Skeletal dysplasia is another defining feature of SDS. Dysplasia can vary greatly in presentation, but is estimated to occur in more than 75% of patients with SDS 1, and may commonly resemble other dysplasias. Short stature is also very common; approximately 33% of patients with SDS are below the 3rd percentile for height and weight 6. Affected individuals may have problems with proper bone formation and growth, which may lead to decreased bone mineral density and increased risk of pathologic fracture 1.

Clinical diagnosis of SDS may be based off findings of pancreatic exocrine dysfunction wit hematologic abnormality. Sequencing of the *SBDS* gene is confirmatory if abnormal. However, approximately 10% of patients with SDS will have normal results of SBDS sequencing 7. This combination of pancreatic exocrine dysfunction and bone marrow failure prompted a working diagnosis of Shwachman‐Diamond syndrome in our patient. However, his presentation is atypical and has defied characterization by comprehensive molecular and cytogenetic evaluation.

It is therefore possible that this patient suffers from a novel syndrome which shows phenotypic overlap with SDS. Interest continues to be focused within the regions of homozygosity (ROH), although no concerning variants were found within these regions, and review of gene map did not yield compelling candidate genes.

This case underscores the difficulty in diagnosis of bone marrow failure disorders, as the presentation of disease can be inconsistent, with complex and ever‐expanding genetic etiologies. Many patients such as this one who develop bone marrow failure do not fall neatly into a previously described category. Additional studies are needed to better characterize such “bone marrow failure, not otherwise specified” patients. Diagnosis in cases of bone marrow failure is not solely of academic interest, as each disorder may have its own associated risk and indications for transplant. As an example, patients with SDS are at increased risk for development of MDS and AML if they are not transplanted and have known sensitivity to typical bone marrow preparative regimens 8. For our patient, the specific risk is unknown and as such the decision to transplant has been complicated, even with benefit of a multidisciplinary team including hematology‐oncology, gastroenterology, endocrinology, genetics, and orthopedics 9.

## Conclusion

Bone marrow failure in infants can be caused by several molecular etiologies. Although our arsenal of tools to interrogate these disorders continues to expand, in some cases we may not have a clear molecular characterization in patients who have progressive disease. Although not ideal, in some of these cases definitive treatment such as bone marrow transplant may be required in the absence of a molecular diagnosis which may complicate treatment and prognosis. Continued investigation into the causes of bone marrow failure in pediatric patients is indicated to close gaps in our knowledge.

## Authorship

MCF: wrote the majority of the discussion and body of the manuscript. ETR: contributed a significant amount of editing and re‐writing of the document, contributed information regarding genetic work‐up and testing for this patient. CKL: wrote the majority of this patient's presentation as well as provided the figures, contributed significant editing and re‐writing of the document.

## Conflict of Interest

The authors have no conflicts of interest to disclose regarding this clinical report.
